# Supplementary material for: Cross-sectional survey among professionals on communication and mental health care for asylum seeking and refugee minors in Germany
Source: Commun Med (Lond). 2026 Feb 4;6:137. doi: 10.1038/s43856-026-01415-x (PMC12979618; doi:10.1038/s43856-026-01415-x)
Supplement: Supplementary file 2 — Supplemental Information [file 43856_2026_1415_MOESM2_ESM.pdf]

Supplement 1:

SAVE-KID: Survey on the Care of Refugee Children and Adolescents in Germany

Welcome to this online survey on the medical care of asylum seeking and refugee minors (ASRM) in Germany. As part of our research, we aim to explore two major areas in detail. The first focuses on infectious disease management, including the implementation of an infectious disease screening. In the second section addresses your experiences with mental health problems in ASRM. The survey pertains to individuals with recent refugee histories who have arrived in Germany within the last two years. We aim to gather insights from a wide range of stakeholders, who, in their professional or voluntary roles, are in contact with children, adolescents, and families with refugee histories. Therefore, the initial questions will ask about your working field. Please feel free to share the invitation link. This study is an online-only survey. Participation is voluntary and pseudonymized. No personally identifiable information will be collected. Completing the questionnaire will take approximately 10-15 minutes. We kindly ask you to complete the entire questionnaire. Please participate only once.

A. Demographic Data

At the beginning of the survey, we would like to ask for some demographic data.

a. How old are you? [___]
b. To which gender do you feel you belong?
   i. Female
   ii. Male
   iii. Other

> If A.b.ii. “Other”:

c. To which social gender do you feel you belong? Please provide the most appropriate description: [___]

B. General Information

Thank you for supporting us with your knowledge and experience in this survey. Please always choose the most appropriate response option in the following questions.

a. Please select your professional role in the care of individuals with refugee histories:
   i. Physician
   ii. Staff in a medical clinic
   iii. Interpreter
   iv. Social worker
   v. Volunteer in refugee care
   vi. Employee of a municipality/regional government office
   vii. Staff in a refugee shelter
   viii. Other professional group

> If B.a.viii. “Other professional group”:

b. Please provide the most appropriate description of your professional role: [___]

> If B.a.i. “Physician”:

c. Please select your medical specialty or the specialty you are pursuing:
   i. Pediatrics and Adolescent Medicine
   ii. General / Family Medicine
   iii. Internal Medicine
   iv. Obstetrics and Gynecology

    v. Dermatology and Venereology
    vi. Child and Adolescent Psychiatry
    vii. Other medical specialty

> If B.a.vii. “Other medical specialty”:

d. Please indicate your medical specialty: [___]

> If B.a.ii. “Staff in medical clinic”:

e. Please select your professional role in the medical clinic:
    i. Pediatric nurse
    ii. Nurse
    iii. Medical assistant
    iv. Paramedic
    v. Research assistant
    vi. Other professional group:

> If B.e.vi. “Other professional group”:

f. Please provide the most appropriate description of your professional role: [___]

g. Please indicate your place of work:
    i. (State) reception center
    ii. Municipal or communal refugee accommodation
    iii. Medical practice
    iv. University hospital
    v. Non-university hospital
    vi. Municipal administration
    vii. Supra-regional administration / government district office
    viii. Cross-institutional / independent
    ix. Other workplace

If B.g.x. “Other workplace”:

h. Please provide the most appropriate description of your workplace: [___]

i. I have been working in the above role for ... years. [___]
j. I work in the above role...
    i. Professionally (i.e., you receive payment for the activity, including part-time or proportionate employment)
    ii. Voluntarily (i.e., no monetary compensation, possibly a trainer allowance, expenses)
k. In which federal state is your place of work located?
    i. Baden-Württemberg
    ii. Bavaria
    iii. Berlin
    iv. Brandenburg
    v. Bremen
    vi. Hamburg
    vii. Hesse
    viii. Mecklenburg-Vorpommern
    ix. Lower Saxony
    x. North Rhine-Westphalia
    xi. Rhineland-Palatinate
    xii. Saarland
    xiii. Saxony
    xiv. Saxony-Anhalt

    xv. Schleswig-Holstein
    xvi. Thuringia
l. In your aforementioned role, how many individuals with refugee histories* have you been in contact over the past three months? [___]
   (*individuals who have fled to Germany within the last two years, including those in ongoing asylum procedures)
m. How many of these were asylum seekers*? [___]
   (*individuals in ongoing asylum procedures)
n. How many of these were ASRM*? [___]
   (*children and adolescents who have fled to Germany within the last two years, including those in ongoing asylum procedures)

>> If B.g.i. “(State) reception center” or ii. “Municipal or communal refugee accommodation”:

C. Workplace

Thank you for this initial information. We would like to explore your workplace in more detail.

a. How many individuals live in your facility at the same time? Please estimate the average of the past three months. [___]
b. How many of them are ASRM? Please estimate the average of the past three months. [___]
c. How many ASRM have you interacted in your role over the past three months? [___]
d. Does your workplace offer medical consultations?
    i. Yes
    ii. No
    iii. Unknown

>> If C.d.i. “Yes”:

e. How often do these medical consultations take place?
    i. Daily
    ii. Weekly
    iii. Monthly
    iv. Less frequently
    v. Unknown
f. Which professional groups are represented in the medical consultation? (Multiple choice)
    i. General practitioners
    ii. Internists
    iii. Pediatricians
    iv. Nurses
    v. Interpreters
    vi. Psychologists
    vii. Midwives
    viii. Other professional groups
    ix. Unknown

> If C.f.viii. “Other professional groups”:

g. Please specify the other professional groups: [___]

> If C.d.ii. „No“:

h. Do you know why consultations are not taking place?
    i. Yes
    ii. No

> If C.h.i. „Yes":

i. What are the reasons for not holding a consultation? (Multiple choice)
    i. No need
    ii. Staff shortage
    iii. Referral to a clinic if necessary
    iv. Referral to a general practitioner if necessary
    v. No cost coverage
    vi. Other reasons:

> If C.i.vi. "Other reasons":

j. Please specify the other reasons: [___]

>> If B.g.iii. "Medical practice":

D. Workplace

Thank you for this initial information. We would like to explore your workplace in more detail.

a. You work in a medical practice. What is the practice's focus of care?
    i. General practitioner care
    ii. Pediatric and adolescent care
    iii. Specialist medical care
b. Approximately how many patients are treated in your practice* over the past three months? [___]
(*for group practices, please specify per chair/seat)
c. Approximately how many refugee patients* are treated in your practice** over the past three months? [___]
(*individuals who have fled to Germany within the last two years, including those in ongoing asylum procedures; **for group practices, please specify per chair/seat)
d. Approximately how many asylum seekers* are treated in your practice** over the past three months? [___]
(*individuals in ongoing asylum procedures, in general cost coverage provided by the social welfare office; **for group practices, please specify per chair/seat)
e. Approximately how many ASRM* are treated in your practice over the past three months? [___]
(*children and adolescents who have fled to Germany within the last two years, including those in ongoing asylum procedures)

>> If B.g.iv. "University hospital" or D.g.v. "Non-university hospital Medical practice":

E. Workplace

Thank you for this initial information. We would like to explore your workplace in more detail.

a. You work in a hospital. Approximately how many refugee patients* have you treated over the past three months? [___]
(*individuals who have fled to Germany within the last two years, including those in ongoing asylum procedures)
b. Approximately how many asylum seekers* have you treated over the past three months? [___]
(*individuals in ongoing asylum procedures, in general cost coverage provided by the social welfare office)
c. Approximately how many ASRM* are treated in your practice per quarter? [___]
(*children and adolescents who have fled to Germany within the last two years, including those in ongoing asylum procedures)

F. **Communication**

a. How often do communication problems arise in the medical care of refugee patients?
   i. Always
   ii. Very frequently
   iii. Frequently
   iv. Occasionally
   v. Rarely
   vi. Never
   vii. Unknown
b. How often do these communication problems impair adequate medical treatment?
   i. Always
   ii. Very frequently
   iii. Frequently
   iv. Occasionally
   v. Rarely
   vi. Never
   vii. Unknown
c. How often do you use the following communication aids?

	Always	Very frequently	Frequently	Occasionally	Rarely	Never
In-person interpreter	○	○	○	○	○	○
Remote video interpreter	○	○	○	○	○	○
Over-the-phone interpreter	○	○	○	○	○	○
Staff members	○	○	○	○	○	○
Relatives	○	○	○	○	○	○
Online tools (i.e. Google translate)	○	○	○	○	○	○

d. Please list any other communication aids you use in your practice: [___]
e. How often do you find yourself without a communication aid when you need one??
   i. Always
   ii. Very frequently
   iii. Frequently
   iv. Occasionally
   v. Rarely
   vi. Never
   vii. Unknown

>> If B.a.i. „Physician":

G. **Infectious Disease Care**

In the following section, we focus on infectious disease screening for ASRM. We would like to know how your practice or department proceeds.

a. Do you conduct vaccinations or infection screening in refugee children and adolescents?
   i. Yes
   ii. No

> If I.a.ii. „No“:

b. If no vaccinations or infection screenings are conducted, where are these measures carried out? (Multiple choice)
   i. Referral to a pediatric clinic
   ii. Referral to a pediatrician
   iii. Referral to a general practitioner
   iv. No structured approach
   v. Already done in advance
   vi. Unknown

/ End of Section I.

>> If I.a.i. „Yes“:

c. Do you conduct other screening measures in ASRM?
   i. Yes
   ii. No

> If I.c.i. „Yes“:

d. What other screening measures do you conduct? (Multiple choice)
   i. Hearing screening
   ii. Vision screening
   iii. Developmental status
   iv. Assessment of relevant psychological conditions or stress factors
   v. Other measures

> If I.d.i. „Other measures“:

e. What other screening measures do you conduct? [___]

f. Do you apply specific measures for infection screening in ASRM under 15 years?
   i. Yes
   ii. No

> If I.f.i. „Yes“:

g. What methods do you use for infection screening in ASRM under 15 years? (Multiple choice)
   i. Anamnesis
   ii. Physical examination
   iii. Infection serologies
   iv. Quantiferon test
   v. Tuberculin skin test
   vi. Chest X-ray
   vii. Blood count
   viii. Clinical chemistry (e.g., GOT, GPT, creatinine)
   ix. Stool examination for parasites
   x. Other examinations

> If I.g.x. „Other examinations“:

h. What other examinations do you conduct? [___]

i. Do you apply specific measures for infection screening in ASRM over 15 years?
   i. Yes
   ii. No

> If I.i.i. „Yes“:

j. What methods do you use for infection screening in ASRM over 15 years? (Multiple choice)
   i. Anamnesis
   ii. Physical examination
   iii. Infection serologies
   iv. Quantiferon test
   v. Tuberculin skin test
   vi. Chest X-ray
   vii. Blood count
   viii. Clinical chemistry (e.g., GOT, GPT, creatinine)
   ix. Stool examination for parasites
   x. Other examinations

> If I.j.x. „Other examinations“:

k. What other examinations do you conduct? [___]

l. Do you regularly use specific infection serologies in standard screenings?
   i. Yes
   ii. No

> If I.l.i. „Yes“:

m. Which infection serologies do you use in standard screenings? (Multiple choice)
   i. HIV
   ii. Hepatitis A
   iii. Hepatitis B
   iv. Hepatitis C
   v. Mumps
   vi. Measles
   vii. Rubella
   viii. Varicella
   ix. Tetanus
   x. Diphtheria
   xi. Schistosoma
   xii. Trypanosoma
   xiii. Other serologies

> If I.m.iiix. „Other serologies“:

n. What other serologies do you use as part of standard screenings? [___]

o. Do you use specific infection serologies in presence of specific risk factors?
   iii. Yes
   iv. No

> If I.o.i. „Yes“:

p. Which infection serologies do you use in presence of specific risk factors? (Multiple choice)
   i. HIV
   ii. Hepatitis A
   iii. Hepatitis B
   iv. Hepatitis C

v. Mumps
vi. Measles
vii. Rubella
viii. Varicella
ix. Tetanus
x. Diphtheria
xi. Schistosoma
xii. Trypanosoma
xiii. Other serologies

> If I.p.iiix. „Other serologies“:

q. What other serologies do you use in presence of specific risk factors? [___]

> If I.m.vi. “Measles” or I.p.vi. “Measles”:

r. What is the reason for determining measles serologies?
   i. Documentation of immunity status for preschool or school
   ii. Evaluate immunity status before vaccination
   iii. Documentation of vaccination efficacy
   iv. Other reason

> If I.r.iv. “Other reason”:

s. What are other reasons for determining measles serologies? [___]

t. Are you offering vaccinations to ASRM?
   i. Yes
   ii. No

> If I.t.i. „Yes“:

u. Which vaccinations do you offer to ASRM?
   i. Diphtheria, Tetanus, and Pertussis vaccine
   ii. IInactivated Poliovirus Vaccine
   iii. Haemophilus influenzae type b vaccine
   iv. Measles, Mumps, and Rubella vaccine
   v. Varicella-Zoster Virus vaccine (known as the chickenpox vaccine)
   vi. Pneumococcal vaccine
   vii. Rotavirus vaccine
   viii. Hepatitis A vaccine
   ix. Hepatitis B vaccine
   x. Meningococcal serotype C vaccine
   xi. Meningococcal serotypes A, C, W, and Y vaccine
   xii. Human Papillomavirus vaccine
   xiii. COVID-19 vaccine
   xiv. Influenza (flu) vaccine
   xv. Additional vaccinations

> If I.u.xv. “Additional vaccination”:

v. Which additional vaccinations do you offer to ASRM? [___]

w. Do you document verbal information in the official vaccination record?
   i. Yes
   ii. No

x. Do you know the guideline and recommendation of the German Society for Pediatric Infectious Diseases (DGPI) on the care and screening in ASRM in Germany?
   i. Yes

ii. No

> If I.x.i. “Yes”:

y. How often are you able to implement these recommendations in practice?
   i. Always
   ii. Very frequently
   iii. Frequently
   iv. Occasionally
   v. Rarely
   vi. Never
   vii. Unknown

z. Do you (additionally) follow other guidelines or recommendations?
   i. Yes
   ii. No

> If I.z.i. “Yes”:

aa. Which other recommendations or guidelines do you follow? [___]

H. Mental health

You’ve reached the final section of the survey. In this part, we aim to explore behavioral and mental health problems among ASRM in Germany, as well as their access to mental health care.

a. Please indicate how frequently you encounter the following behavioral and mental health problems among ASRM.

	Always	Very frequently	Frequently	Occasionally	Rarely	Never	Unknown
Aggressiveness towards oneself	○	○	○	○	○	○	○
Aggressiveness towards others	○	○	○	○	○	○	○
Sadness	○	○	○	○	○	○	○
Apathy	○	○	○	○	○	○	○
Irritability	○	○	○	○	○	○	○
Lack of concentration	○	○	○	○	○	○	○
Tiredness	○	○	○	○	○	○	○
Restlessness	○	○	○	○	○	○	○
Impulsiveness	○	○	○	○	○	○	○
Hyperactivity	○	○	○	○	○	○	○
Lonerism	○	○	○	○	○	○	○
Sleep problems	○	○	○	○	○	○	○
Nightmares	○	○	○	○	○	○	○
Recurrent headaches	○	○	○	○	○	○	○
Recurrent abdominal pain	○	○	○	○	○	○	○
Drug and alcohol use	○	○	○	○	○	○	○

b. If you wish, please list any other behavioral or mental health problems that you have observed: [___]
c. Approximately, how many ASRM with one or more of these behavioral or mental health problems have you been in contact with over the past three months? (as integer) [___]
d. To your knowledge, how many of these ASRM have been referred for further follow-up care due to their behavioral or mental health problems? (as integer) [___]
e. In your opinion, are you able to meet the needs of these ASRM with behavioral or mental health problems?
    i. Always
    ii. Very frequently
    iii. Frequently
    iv. Occasionally
    v. Rarely
    vi. Never
    vii. Unknown
f. Do you ever feel overwhelmed when working with ASRM who have behavioral or mental health problems?
    i. Always
    ii. Very frequently
    iii. Frequently
    iv. Occasionally
    v. Rarely
    vi. Never
    vii. Unknown
g. Do you have professional training in working with minors who have behavioral or mental health problems?
    i. Yes
    ii. No
h. At your workplace, ere ASRM routinely screened for psychological stress and mental health problems?
    i. Always
    ii. Very frequently
    iii. Frequently
    iv. Occasionally
    v. Rarely
    vi. Never
    vii. Unknown

> If J.h.i./ii./iii./iv./v. “Always / very frequently / frequently / occasionally /rarely”:

i. Which screening methods are used? [___]

j. At your workplay, are behavioral problems in ASRM routinely documented?
    i. Always
    ii. Very frequently
    iii. Frequently
    iv. Occasionally
    v. Rarely
    vi. Never
    vii. Unknown
k. Do you have adequate resources* available to further assessand care for minors with behavioral or mental health problems?

(*By resources, we mean specialized staff or facilities, such as social pediatric centers (SPC), pediatricians with respective experience, educators, psychologists, psychiatrists, etc.)

  i. Always
  ii. Very frequently
  iii. Frequently
  iv. Occasionally
  v. Rarely
  vi. Never
  vii. Unknown

l. How do you experience the accessibility of these resources for ASRM (compared to minors who have grown up in Germany)?
  i. Significantly easier
  ii. Somewhat easier
  iii. Similarly easy / difficult
  iv. Somewhat more difficult
  v. Significantly more difficult
  vi. Unknown

m. In your opinion, what makes access to resources more difficult? (Multiple choice)
  i. Cost coverage not guaranteed
  ii. Lack of available appointments
  iii. Lack of available interpreters
  iv. Resources spatially not accessible for ASRM and their families
  v. Resources not/poorly accepted by ASRM and their families for scheduling reasons
  vi. Resources not/poorly accepted by ASRM and their families for cultural reasons
  vii. Resources / Facilities reject the treatment of ASRM
  viii. Other obstacles

n. In your experience, what other barriers make access more difficult? [___]

o. In your experience, what factors facilitate access to the resources mentioned? [___]
